# Distribution and ultrastructural localization of the glucagon-like peptide-1 receptor (GLP-1R) in the rat brain

**DOI:** 10.1007/s00429-020-02189-1

**Published:** 2020-12-20

**Authors:** Erzsébet Farkas, Anett Szilvásy-Szabó, Yvette Ruska, Richárd Sinkó, Morten Grønbech Rasch, Thomas Egebjerg, Charles Pyke, Balázs Gereben, Lotte Bjerre Knudsen, Csaba Fekete

**Affiliations:** 1 Laboratory of Integrative Neuroendocrinology, Institute of Experimental Medicine, Budapest, 1083 Hungary; 2 Novo Nordisk A/S, 2760 Måløv, Denmark; 3 Department of Medicine, Division of Endocrinology, Diabetes and Metabolism, Tupper Research Institute, Tufts Medical Center, Boston, MA 02111 USA

**Keywords:** GLP-1 receptor, Axonal localization, Distribution, Immunohistochemistry, Electron microscopy

## Abstract

Glucagon-like peptide-1 (GLP-1) inhibits food intake and regulates glucose homeostasis. These actions are at least partly mediated by central GLP-1 receptor (GLP-1R). Little information is available, however, about the subcellular localization and the distribution of the GLP-1R protein in the rat brain. To determine the localization of GLP-1R protein in the rat brain, immunocytochemistry was performed at light and electron microscopic levels. The highest density of GLP-1R-immunoreactivity was observed in the circumventricular organs and regions in the vicinity of these areas like in the arcuate nucleus (ARC) and in the nucleus tractus solitarii (NTS). In addition, GLP-1R-immunreactive (IR) neuronal profiles were also observed in a number of telencephalic, diencephalic and brainstem areas and also in the cerebellum. Ultrastructural examination of GLP-1R-immunoreactivity in energy homeostasis related regions showed that GLP-1R immunoreactivity is associated with the membrane of perikarya and dendrites but GLP-1R can also be observed inside and on the surface of axon varicosities and axon terminals. In conclusion, in this study we provide a detailed map of the GLP-1R-IR structures in the CNS. Furthermore, we demonstrate that in addition to the perikaryonal and dendritic distribution, GLP-1R is also present in axonal profiles suggesting a presynaptic action of GLP-1. The very high concentration of GLP-1R-profiles in the circumventricular organs and in the ARC and NTS suggests that peripheral GLP-1 may influence brain functions via these brain areas.

**Supplementary Information:**

The online version contains supplementary material available at 10.1007/s00429-020-02189-1.

## Introduction

Glucagon-like peptide-1 (GLP-1) is an incretin hormone (Baggio and Drucker 2007). It is derived from the posttranslational processing of proglucagon (Baggio and Drucker 2007). This prohormone is synthesized by three cell populations, the neuroendocrine L cells of the intestinal mucosa, the ß cells of the pancreatic Langerhans islands and in a neuronal population located in the nucleus tractus solitarii (NTS) and intermediate reticular nucleus of the medulla oblongata (Muller et al. 2019). GLP-1 is produced primarily in the gut and the brain as its production requires PC1/3 catalyzed posttranslational processing of proglucagon (Muller et al. 2019). Stress and type 2 diabetes, however, upregulate PC1/3 expression in pancreatic α cells enabling GLP-1 production in this tissue (Chen et al. 2018). GLP-1 has a critical role in the regulation of energy and glucose homeostasis (Andersen et al. 2018), and exerts its effects via the GLP-1 receptor (GLP-1R). It decreases circulating glucose levels by slowing down the gastric emptying, and by stimulating insulin production and inhibiting glucagon secretion (Andersen et al. 2018). Furthermore, GLP-1 has a potent inhibitory effect on food intake and can decrease the hedonic value of food and the motivation to eat (Andersen et al. 2018; Hayes and Schmidt 2016). These properties have made GLP-1R an important drug target in the field of type 2 diabetes and obesity. Long-acting GLP-1R agonists, liraglutide, semaglutide and dulaglutide have been marketed for the treatment of type 2 diabetes and have been shown to also provide cardiovascular risk reduction (Gerstein et al. 2019; Marso et al. 2016; Davies et al. 2017), while liraglutide is approved for the treatment of obesity (Pi-Sunyer et al. 2015). Semaglutide has completed phase 3 for obesity. Studies using a variety of histology-based methods have convincingly demonstrated the widespread distribution of GLP-1R in the central nervous system (CNS) (Merchenthaler et al. 1999; Cork et al. 2015; Heppner et al. 2015; Jensen et al. 2018). Little information is available, however, about the distribution of the GLP-1R protein at the ultrastructural level in brain tissue. In addition, no previous description of GLP-1R immunoreactivity in the rat CNS has been published.

Due to the very short half-life, the ability of endogenous GLP-1 to enter the brain has been debated, but the clinically used, long-acting GLP-1R agonists (liraglutide and semaglutide) have been proven to enter the brain of rodents and in these animals, can bind to central neuronal groups relevant for the weight effects observed in the clinic (Secher et al. 2014; Gabery et al. 2020). Thus, it is important to understand which neuronal populations can be influenced by the administration of these compounds.

We, therefore, mapped the distribution of the GLP-1R-immunoreactive (IR) profiles in the rat brain and examined the subcellular localization of this receptor in energy homeostasis related brain regions using immune-electron microscopy.

## Material and methods

### Animals

Adult, male Sprague–Dawley rats weighing 250–280 g and wild type C57/BL/6 and GLP-1R KO mice were used.

Animals were housed under standard environmental conditions (lights on between 06.00 and 18.00 h, temperature 22 ± 1 °C, mouse chow and water ad libitum). All experimental protocols were reviewed and approved by the Animal Welfare Committee at the Institute of Experimental Medicine. All animal experiments were performed in accordance with relevant guidelines of Novo Nordisk.

Rats were anaesthetized with a mixture of ketamine and xylazine (ketamine 50 mg/kg, xylazine 10 mg/kg body weight, i.p.) and were perfused transcardially with 30 ml 0.01 M phosphate-buffered saline pH 7.4 (PBS), followed by 150 ml 3% paraformaldehyde (Science Services GmbH, München, Germany) plus 1% acrolein (Merck KGaA, Darmstadt, Germany) in 0.1 M phosphate buffer pH 7.4 (PB). Anaesthetized mice were perfused transcardially with 10 ml 0.01 M phosphate-buffered saline pH 7.4 (PBS), followed by 40 ml mixture of 3% paraformaldehyde (Science Services GmbH, München, Germany) and 1% acrolein (Merck KGaA, Darmstadt, Germany) in 0.1 M phosphate buffer pH 7.4 (PB). The brains were rapidly removed. For light microscopy (*N* = 6 rats; *N* = 3 WT C57BL/6 mice; *N* = 2 GLP-1R KO mice), the brains were cryoprotected in 30% sucrose in 0.01 M PBS overnight at room temperature (RT). For electron microscopy (*N* = 3 rats; *N* = 3 C57BL/6 mice), the brains were postfixed in 4% PFA in 0.1 M PB pH 7.4.

### Antibodies

The light and electron microscopic studies on rat tissues were performed using the mouse monoclonal anti-GLP-1R antibody (Clone 7F38A2; 0.5 µg/ml, Novo Nordisk A/S) (Jensen et al. 2018). To determine the specificity of the antibody, immunocytochemistry was performed on brain sections of WT and GLP-1R knockout mice using the same protocol as the one used for rat sections. Endogenous IgGs are abundant in the circumventricular organs and in the arcuate nucleus of the mouse brain (Fabian and Ritchie 1986) and therefore, immunostaining using mouse primary antibody results in non-specific labeling in these regions due to the reaction of endogenous IgG with the secondary antibody. To validate the specificity of the monoclonal anti-GLP-1R antibody in these brain regions, a rabbitized version of the antibody was generated using recombinant technology, whereby the heavy and light chain variable regions from the mouse monoclonal anti-GLP-1R antibody were grafted onto a rabbit IgG to generate a rabbit monoclonal version of clone 7F38A2 (detailed in Supplemental Fig. 1 and as described earlier (Bhatti et al. 2019)).

### Single-labeling immunocytochemistry for detection of GLP-1R-immunoreactivity

Serial, 25 µm thick coronal sections through the whole brain were cut on a freezing microtome (Leica Microsystems, Vienna, Austria). The sections were washed with PBS and treated with 1% sodium borohydride (Cat. #45882, Merck) in 0.1 M PB for 30 min and then treated with 0.5% Triton X-100 (Cat. # T9284, Merck) and 0.5% H_2_O_2_ (Cat. # 95321, Merck) in 0.01 M PBS for 15 min. Nonspecific antibody binding was blocked with treatment in 2% normal horse serum (NHS) in PBS for 20 min, and then the rat sections were placed in mouse monoclonal anti-GLP-1R antibody (Clone 7F38A2; 0.5 µg/ml, Novo Nordisk A/S) while the mouse sections were immersed in either mouse monoclonal anti-GLP-1R antibody (Clone 7F38A2; 0.5 µg/ml) or in rabbitized monoclonal antibody against GLP-1R (0.016 µg/ml) diluted in serum diluent (2% NHS + 0.2% sodium azide in PBS) for 2 days at 4 °C. After rinsing in PBS, the sections were incubated in either biotinylated donkey anti-mouse IgG (Cat. # 715-065-151, Jackson ImmunoResearch Europe Ltd, Ely, UK) or biotinylated donkey anti-rabbit IgG (Cat. # 711-065-152, Jackson ImmunoResearch Europe Ltd, Ely, UK) diluted at 1:500 in serum diluent at room temperature. After washing in PBS, the sections were treated with avidin–biotin-peroxidase complex (ABC Elite, Cat. # PK-6100, 1:1000, Vector Laboratories Ltd, Peterborough, UK) for an hour and the GLP-1R-immunoreactivity was developed in Ni-DAB developer (0.05% diaminobenzidine and 0.15% nickel ammonium sulfate 0.005% H_2_O_2_ in 0.05 M Tris buffer, pH 7.6). The sections were mounted onto glass slides and coverslipped with DPX mounting medium (Cat. # 06522, Merck). Images were taken using a Zeiss AxioImager M1 microscope equipped with AxioCam MRc5 digital camera (Carl Zeiss Inc., Darmstadt, Germany). To facilitate the mapping of GLP-1R-IR structures, mosaic images were taken. The maps were drawn in CorelDraw Graphics Suite (Corel Corporation, Ottawa, Canada) based on these images and the plates of the Atlas of Paxinos and Watson (Paxinos and Watson 2013). One set of native sections from each brain was Nissl stained to facilitate the mapping. Every fourth section of three rat brains were analyzed by two independent observers.

### Tissue preparation for ultrastructural studies

Serial, 25 µm thick coronal sections were cut on a Leica VT 1000S vibratome (Leica Microsystems, Vienna, Austria) through the whole brains. The sections were collected in 0.1 M PBS (pH 7.4). The sections were transferred into anti-freeze solution (30% ethylene glycol (Cat. # 297, VWR Chemicals); 25% glycerol (Cat. # 24388.295, VWR Chemicals); 0.05 M PB) and stored at − 20 °C until their use for immunohistochemistry.

The sections were then washed with PBS and treated with 1% sodium borohydride in 0.1 M PB for 30 min and then with 0.5% H_2_O_2_ in PBS for 15 min. The sections were cryoprotected in 15% sucrose in PBS for 30 min at room temperature and in 30% sucrose in PBS overnight at 4 °C, and then quickly frozen over liquid nitrogen and thawed. The freezing–thawing cycle was repeated three times to improve antibody penetration.

The pretreated sections were incubated in 2% normal horse serum (NHS, diluted in 0.1 M PBS) for 20 min. Then, the rat sections were placed in mouse monoclonal anti-GLP-1R (0.5 µg/ml, clone 7F38A2) and the mouse sections were placed in rabbitized monoclonal antibody against GLP-1R (0.016 µg/ml) in serum diluent for 4 days at 4 °C. After rinsing in PBS, the sections were incubated in biotinylated donkey anti-mouse or anti-rabbit IgG diluted at 1:500 in serum diluent overnight at 4 °C followed by treatment in ABC (1:1000). The GLP-1R-immunoreactivity was developed in Ni-DAB developer. The sections were rinsed in 0.2 M sodium citrate (Cat. # S4641, Merck) pH 7.5, and then the immunoreaction was silver intensified with the Gallyas method (Liposits et al. 1984) for 2.5 min. The sections were placed in 0.05% gold-chloride (Cat. # 520918, Merck) for 10 min at room temperature, washed in 0.2 M sodium citrate, pH 7.5, and in 3% sodium-thiosulphate solution for 10 min each at room temperature.

### Embedding and ultrastructural examination of the immunostained sections

Sections were incubated in 1% osmium-tetroxide for 1 h at room temperature and then treated with 2% uranyl acetate in 70% ethanol for 30 min. Following dehydration in an ascending series of ethanol and acetonitrile (Cat. # 360457, Merck), the sections were flat embedded in Durcupan ACM epoxy resin (Cat. # 44610, Merck) on liquid release agent (Cat. # 70880, Electron Microscopy Sciences)-coated slides, and polymerized at 56 °C for 2 days. After polymerization, 60–70 nm thick ultrathin sections were cut with Leica UCT ultramicrotome (Leica Microsystems). The ultrathin sections were mounted onto Formvar-coated, single-slot grids, treated with lead citrate and examined with a JEOL-100 C transmission electron microscope.

## Results

### Antibody validation

**Fig. 1:**
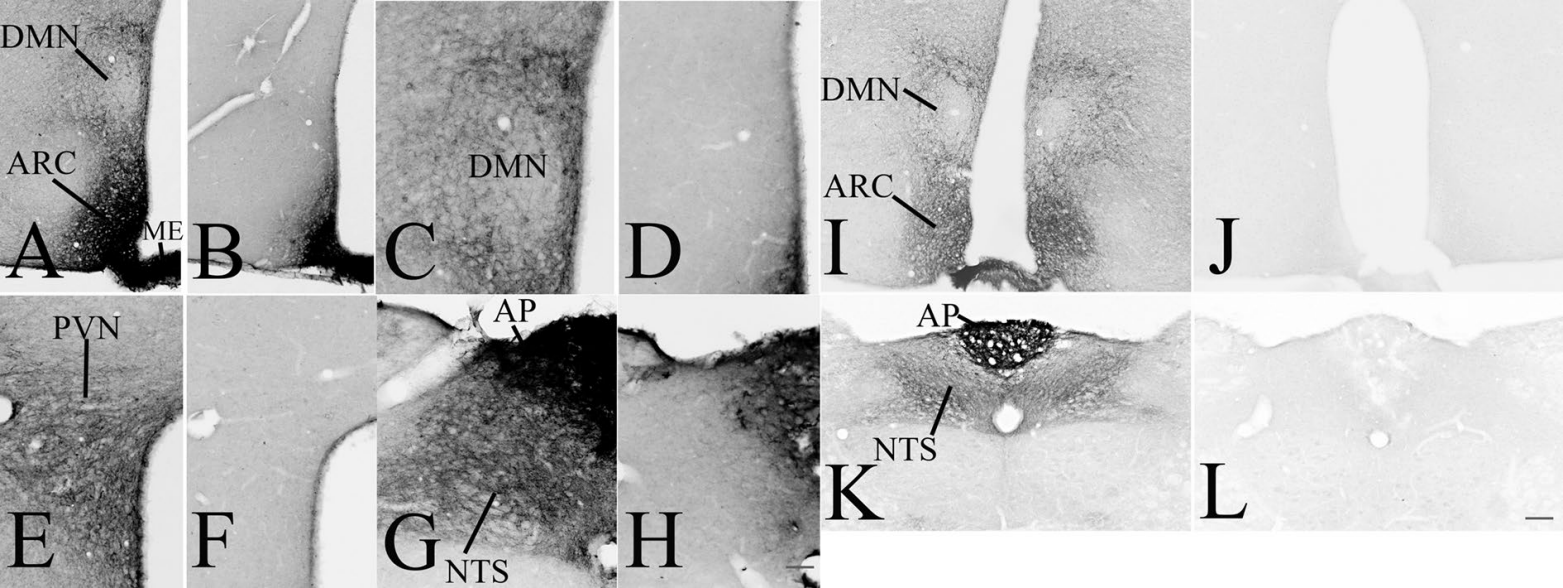
Specificity of the used monoclonal GLP-1R antibody. GLP-1R-immunoreactivity using the mouse monoclonal anti-GLP-1R IgG (**a**, **c**, **e**, **f**), results in similar immunostaining as it was observed in rats, however, the circumventricular organs, like the median eminence (ME) and the area postrema (AP) and parts of the arcuate nucleus (ARC), where mouse IgGs are present (Fabian and Ritchie 1986), are filled with immunoreaction product. In GLP-1R KO mice, the immunoreaction product is absent except the circumventricular organs and the ARC, where the used secondary antibody stains the mouse IgGs (**b**, **d**, **f**, **h**). To prove the specificity of the antibody even in these areas, immunostaining was performed in WT (**i**, **k**) and GLP-1R KO (**j**, **l**) mice using the rabbitized version of the antibody. The immunostaining with the rabbitized antibody in WT mice was highly similar than what was observed in rats, but the immunoreaction product was completely absent when sections of GLP-1R KO mice were stained. Scale bar (shown in **h**): **c**–**h**, 50 µm; (shown in **L**) **a**, **b**, **i**–**l** 100 µm. *DMN* hypothalamic dorsomedial nucleus, *PVN* hypothalamic paraventricular nucleus, *NTS* nucleus tractus solitarii

To determine the specificity of the mouse monoclonal anti-GLP-1R antibody used throughout for mapping GLP-1R in rat brain sections, immunostaining was performed on brain sections of wild type and GLP-1R KO mice. The pattern of immunoreaction was very similar in WT mice (Fig. 1a, c, e, g) as it was observed in rats (Figs. 3, 4, 5, 6, 7 and 8) except in the circumventricular organs and most parts of the arcuate nucleus, which regions were almost completely filled with immunoreaction product in mice. In GLP-1R KO mice, the immunoreaction product was completely absent except in the circumventricular organs and most parts of the arcuate nucleus (Fig. 1b, d, f, h). It is well known that mouse IgG is present in these brain areas that is detected by the used secondary antibody (Fabian and Ritchie 1986). To be able to determine the specificity of the used antibody even in these brain regions, immunostaining was also performed using the rabbitized version of the monoclonal GLP-1R antibody that has identical antigen recognition region as the original mouse monoclonal antibody.

This immunostaining with the rabbitized antibody resulted in a very similar pattern as with the original mouse monoclonal antibody. However, in the circumventricular organs and in the ARC, the almost homogenous NiDAB precipitate was replaced with the labeling of neuronal profiles as it was observed in rats. The immunoreaction with this antibody was completely absent in all brain regions of GLP-1R KO mice demonstrating the specificity of the antibody (Fig. 1k, l).

### Distribution of GLP-1R-immunoreactivity in the rat brain

**Fig. 2:**
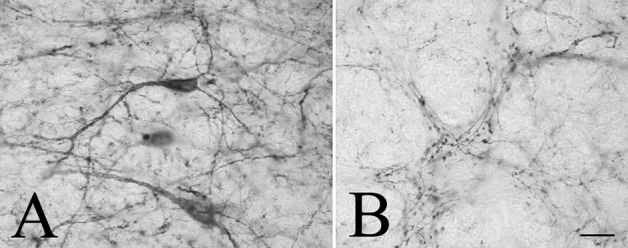
Presence of the GLP-1R-immunoreactivity in both neuronal perikarya and axon like profiles. GLP-1R-immunoreactivity was frequently observed in the brain in neuronal perikarya and dendrites (**a**), but also in varicose axon like profiles (**b**). Scale bar = 20 µm

**Fig. 3:**
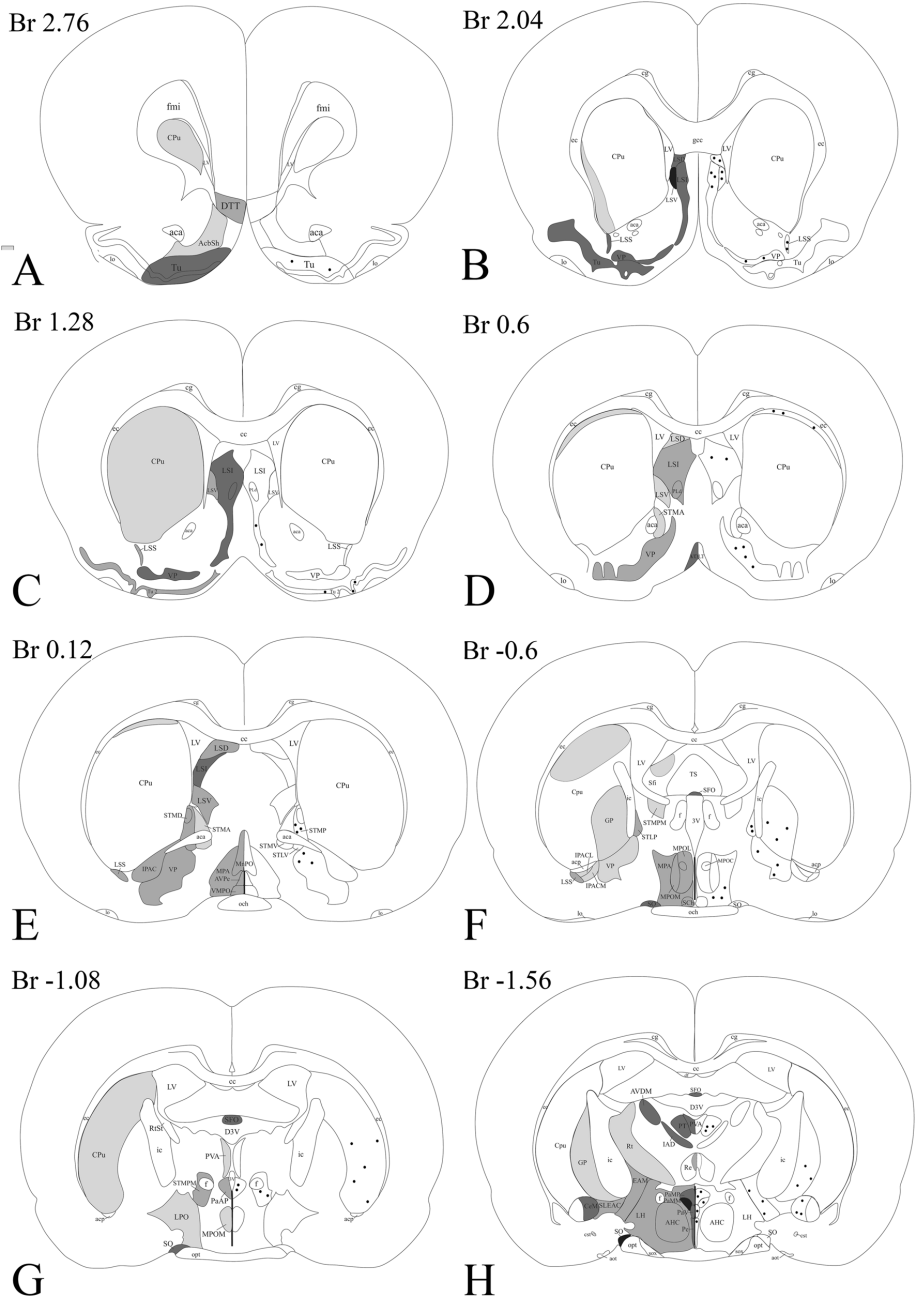

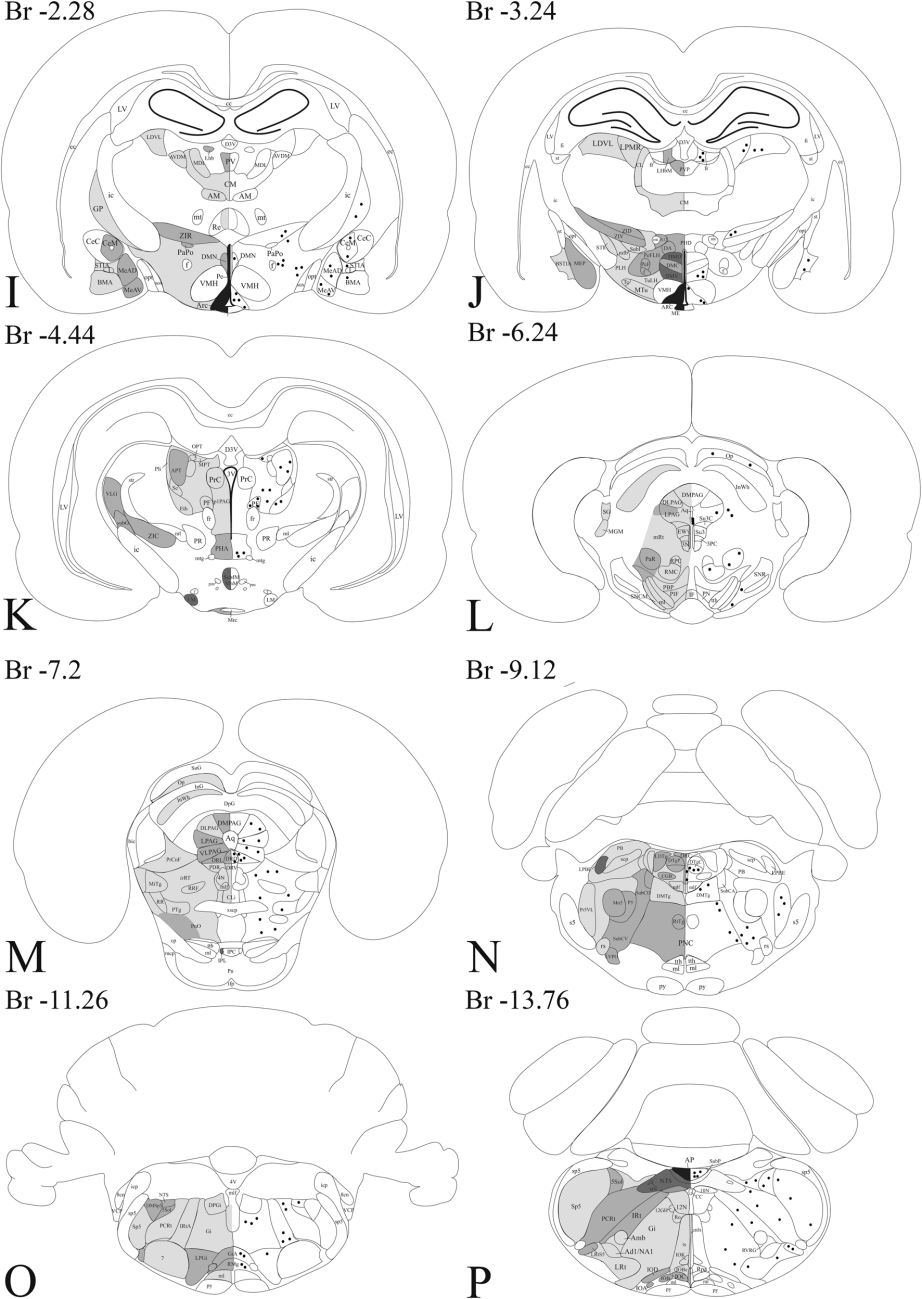
Schematic illustration of the distribution of GLP-1R-IR processes (left side) and perikarya (right side) in the rat brain mapped to plans of the Paxinos and Watson (2013). The darkness of the area corresponds to the density of GLP-1R-IR processes. Dots mark the region where GLP-1R perikarya are present. The distance of the coronal plans from the Bregma is shown above the maps. The data is based on the sections of three rat brains in which the distribution of GLP-1R-immunreactivity was similar.

A widespread distribution of the GLP-1R protein was observed throughout the rostro-caudal extent of the brain. The GLP-1R-immunoreactivity had a similar distribution in the three studied brains. In general, the intensity of the GLP-1R immunoreactivity was the highest in the circumventricular organs including the area postrema, subfornical organ, median eminence and organum vasculosum laminae terminalis (OVLT), and in areas located in the close proximity of the circumventricular organs like the arcuate nucleus (ARC) and the NTS. GLP-1R immunoreactivity was observed in both fibers and cell bodies (Fig. 2). The distribution of GLP-1R-IR fibers and cell bodies is summarized in Tables 1, 2, 3, 4, 5 and 6 and Fig. 3.

**Table 1:** Distribution of the GLP-1R-IR structures in the telencephalon

Brain areas	Processes	Somata	Abbreviation
Dorsal tenia tecta	++		DTT
Olfactory tubercle	++	*	Tu
Lateral stripe of striatum	+		LSS
Lateral septal nucleus intermediate part	+++	*	LSI
Lateral septal nucleus dorsal part	++	*	LSD
Lateral septal nucleus ventral part	++++	*	LSV
Nucleus accumbens schell	+		AcbSH
Ventral Pallidum	++	*	VP
Globus pallidum	++	*	GP
Caudate Putamen	+	*	CPu
Bed nucleus of the stria terminalis	++	**	BNST
Subfornical organ	+++		SFO
Interstitial nucleus of the posterior limb of the anterior commissurae	+		IPAC
Central amygdaloid nucleus medial division	++	**	CeM
Bed nucleus of the stria terminalis, intraamygdaloid division	+	*	BSTIA
Sublenticular extended amygdala medial part	++		SLEAM
Sublenticular extended amygdala central part	++	*	SLEAC
Medial amygdaloid nucleus anterior dorsal part	++	*	MeAD
Medial amygdaloid nucleus anteroventral part	+	*	MeAV
Medial amygdaloid nucleus posterior part	++		MEP
Basomedial amygdaloid nucleus anterior part	+	*	BMA
Intercalated nucleus of the amygdala	+	*	I
Intercalated amygdaloid nucleus main part	+	*	IM

The GLP-1R-immunoreactive areas were ranked into four categories based on the density of immunoreactive fibers from “+”, meaning low level to “++++” meaning “very high” level, and based on the density of immunoreactive perikarya from *meaning low to ***meaning high level

**Table 2:** Distribution of the GLP-1R-IR structures in the thalamus, subthalamus and epithalamus

Brain areas	Processes	Somata	Abbreviation
Mediodorsal thalamic nucleus lateral part	+		MDL
Interanterodorsal thalamic nucleus	+++		IAD
Anteromedial thalamic nucleus	+		AM
Anteroventral thalamic nucleus medial part	+		AVDM
Lateroposterior thalamic nucleus mediorostral part	+		LPMR
Laterodorsal thalamic nucleus ventrolateral part	+		LDVL
Central medial thalamic nucleus	+		CM
Centrolateral thalamic nucleus	+		CL
Parafascicular thalamic nucleus	+	*	PF
Paraventricular thalamic nucleus	++		PV
Paratenial thalamic nucleus	++	*	PT
Reuniens thalamic nucleus	++		Re
Posterior limitans thalamic nucleus	+		PLi
Medial geniculate nucleus medial part	+		MGM
Ventral lateral geniculate nucleus	++		VLG
Ethmoid thalamic nucleus	+	*	Eth
Scaphoid thalamic nucleus	+		Sc
Reticular thalamic nucleus	+		Rt
Lateral Habenular nucleus	+*/*++		LHb
Subincertal nucleus	+		Sub1
Subthalamic nucleus	+		STh
Subgeniculate nucleus	++		SubG
Suprageniculate thalamic nucleus	+		SG
Zona incerta	++	*	ZI

The GLP-1R-immunoreactive areas were ranked into four categories based on the density of immunoreactive fibers from “+”, meaning low level to “++++” meaning “very high” level, and based on the density of immunoreactive perikarya from *meaning low to ***meaning high level

**Table 3:** Distribution of the GLP-1R-IR structures in the hypothalamus

Brain areas	Processes	Somata	Abbreviation
Organum vasculosum laminae terminalis	+++		VOLT
Medial preoptic area	++	*	MPA
Medial preoptic nucleus	++		MPOC
Lateral preoptic area	+		LPO
Ventromedial preoptic nucleus	++		VMPO
Median preoptic nucleus	+++		MnPO
Anteroventral periventric nucleus	++		AVPe
Supraoptic nucleus	+++		SO
Paraventricular nucleus anterior parvocellular subdivision	++	*	PaAP
Paraventricular nucleus magnocellular division	+++	*	PaMM
Paraventricular nucleus medial parvocellular subdivison	+++	*	PaMP
Paraventricular nucleus ventral parvocellular subdivison	+++	**	PaV
Paraventricular nucleus posterior part	++		PaPo
Dorsomedial hypothalamic nucleus compact part	++		DMC
Dorsomedial hypothalamic nucleus dorsal part	+++		DMD
Dorsomedial hypothalamic nucleus ventral part	+++	*	DMV
Anterior hypothalamic area central	++		AHC
Periventricular hypothalamic nucleus	+++	*	Pe
Arcuate nucleus	++++	***	ARC
Dorsal hypothalamic area	++		DA
Lateral hypothalamus	++	*	LH
Perifornical part of lateral hypothalamus	++		PeFLH
Peduncular part of lateral hypothalamus	+		PLH
Posterior hypothalamic area	++	*	PHA
Posterior hypothalamic area dorsal	++		PHD
Medial forebrain bundle	+		mfb
Median eminence	++++		Me
Terete hypothalamic nucleus	+		Te
Tuberal region of lateral hypothalamus	+	*	TuLH
Supramammillary nucleus medial	+++		SuMM
Medial tuberal nucleus	+		MTu
Medial mammillary nucleus	+++		MnM
Lateral mammillary nucleus	+++		LM

The GLP-1R-immunoreactive areas were ranked into four categories based on the density of immunoreactive fibers from “+”, meaning low level to “++++” meaning “very high” level, and based on the density of immunoreactive perikarya from *meaning low to ***meaning high level

**Table 4:** Distribution of the GLP-1R-IR structures in the mesencephalon

Brain areas	Processes	Somata	Abbreviation
Anterior pretectal nucleus	++	*	APT
Interpeduncular nucleus lateral part	+++	*	IPL
Substancia Nigra compact part, medial tier	+		SNCM
Substancia Nigra reticular part		*	SNR
Medial lemniscus	+		ml
Trigeminothalamic tract	+		tth
Parabrachial pigmented nucleus	+*/*++		PBP
Parainterfascicular nucleus	+		PIF
Paranigral nucleus of VTA	+		PN
Ventral tegmental area	+		VTA
Interfascicular nucleus	+		IF
Caudal linear nucleus of the raphe	+		CLi
Retrorubral nucleus	+		RR
Retrorubral field	+		RRF
Pararubral nucleus	++	*	PaR
Red nucleus parvicellular part	+		RPC
Red nucleus magnocellular part	+	*	RMC
Oculomotor nucleus	+		3 N
Oculomotor nucleus parvicellular part	+		3PC
Supraoculomotor periaquaduct	+		SU3
Supraoculomotor cap	+		SU3C
Trochlear nucleus	+		4 N
Edinger –Westphal nucleus	++		EW
Periaqueductal gray dorsolateral part	+	*	DLPAG
Lateral periaqueductal gray	++	*	LPAG
Dorsomedial Periaqueductal gray	+*/*++		DMPAG
Ventrolateral Periaqueductal gray	++	**	VLPAG
Precuneiform nucleus	+		PrCnF
Mesencephalic reticular formation	+		mRT
Isthmic reticular formation	+	*	isRT
Microcellular tegmental nucleus	+		MiTg
Dorsal raphe nucleus lateral part	++	**	DRL
Dorsal raphe nucleus dorsal part	++	**	DRD
Posterodorsal raphe nucleus	+		PDR
Dorsal raphe nucleus ventral part	+		DRV
Optic nerve layer of superior colliculus	+	*	Op
Intermediate white layer of superior colliculus	+		InWh

The GLP-1R-immunoreactive areas were ranked into four categories based on the density of immunoreactive fibers from “+”, meaning low level to “++++” meaning “very high” level, and based on the density of immunoreactive perikarya from *meaning low to ***meaning high level

**Table 5:** Distribution of the GLP-1R-IR structures in the pons

Brain areas	Processes	Somata	Abbreviation
Reticulotegmental nucleus pons	+		RtTg
Locus ceroleus	+		LC
Subcoerulens nucleus alpha part	+		SubCA
Subcoerulens nucleus dorsal part	+		SubCD
Subcoerulens nucleus ventral part	++	**	SubCV
Pedunculopontine tegmental nucleus	+		PTg
Pontine reticular nucleus oral part	+*/*++	*	PnO
Pontine reticular nucleus caudal part	++	*	PNC
Intermediate reticular nucleus alpha	+	*	IRtA
Laterodorsal tegmental nucleus	++	*	LDTg
Dorsal tegmental nucleus central part	++	***	DTgC
Dorsal tegmental nucleus pericentral part	++		DTgP
Dorsomedial tegmental area	+	*	DMTg
Medial longitudional fascicle	+		mlf
Central gray ß part	++		CGB
Parabrachial nucleus	+		PB
Lateral parabrachial nucleus external part	+++		LPBE
Motor trigeminal nucleus	++		Mo5
Principal sensory trigeminal nucleus ventrolateral part	+	*	Pr5VL
Peritrigeminal zone	++		P5
Spinal trigeminal nucleus	+*/*++	*	Sp5
Spinal trigeminus nucleus dorsomedial part	++		DMSp5
Trigeminal-solitary transition zone	++	*	5Sol
Facial nucleus	+		7
Lateral paragigantocellular nucleus	++	*	LPGi
Raphe magnus	++	*	RMg
Lateroventral periolivary nucleus	+		LVPO
Medial lemniscus	+		ml
Dorsal raphe nucleus caudal part	++	*	DRC

The GLP-1R-immunoreactive areas were ranked into four categories based on the density of immunoreactive fibers from “+”, meaning low level to “++++” meaning “very high” level, and based on the density of immunoreactive perikarya from *meaning low to ***meaning high level

**Table 6:** Distribution of the GLP-1R-IR structures in the medulla oblongata

Brain areas	Processes	Somata	Abbreviation
Intermediate reticular nucleus	++	*	IRt
Intermediate reticular nucleus alpha	+	*	IRtA
Gigatocellular reticular nucleus	+	*	Gi
Gigatocellular reticular nucleus alpha	++		GiA
Lateral paragigatocellular nucleus	+		LPGiA
Lateral reticular nucleus	+	*	LRt
Lateral reticular nucleus substrigeminal part	++	*	LRtS5
Dorsal paragigatocellular nucleus	++	*	DPGi
Parapyramidal nucleus of raphe	++		PPy
Parvicellular reticular nucleus	++	*	PCRt
Spinal trigeminal nucleus	+	*	Sp5
Trigeminal-solitary transition zone	++	*	5Sol
Nucleus of the solitary tract	+++		NTS
Area postrema	++++	****	AP
Dorsal motor nucleus of vagus	+++		10 N
Hypoglossal nucleus	+		12 N
Ad1 adrenergic and NA1 noradrenergic region	+		Ad1/NA1
Ambigus nucleus	+		Amb
Rostral Ventral Respiratory group			RVRG
Nucleus of Roller	+		Ro
Hypoglossal nucleus geniohyoid part	+		12GH
Inferior olive, dorsal nucleus	++		IOD
Inferior olive, subnuclei A, B and C of medial nucleus	++		IOA; IOB and IOC
Inferior olive, cap of Kooy	+		IOK
Inferior olive, beta subnucleus	+		IOBe
Tectospinal tract	+		ts
Raphe pallidus nucleus	++		Rpa

The GLP-1R-immunoreactive areas were ranked into four categories based on the density of immunoreactive fibers from “+”, meaning low level to “++++” meaning “very high” level, and based on the density of immunoreactive perikarya from *meaning low to ***meaning high level

#### Distribution of GLP-1R-immunoreactivity in the telencephalon

**Fig. 4:**
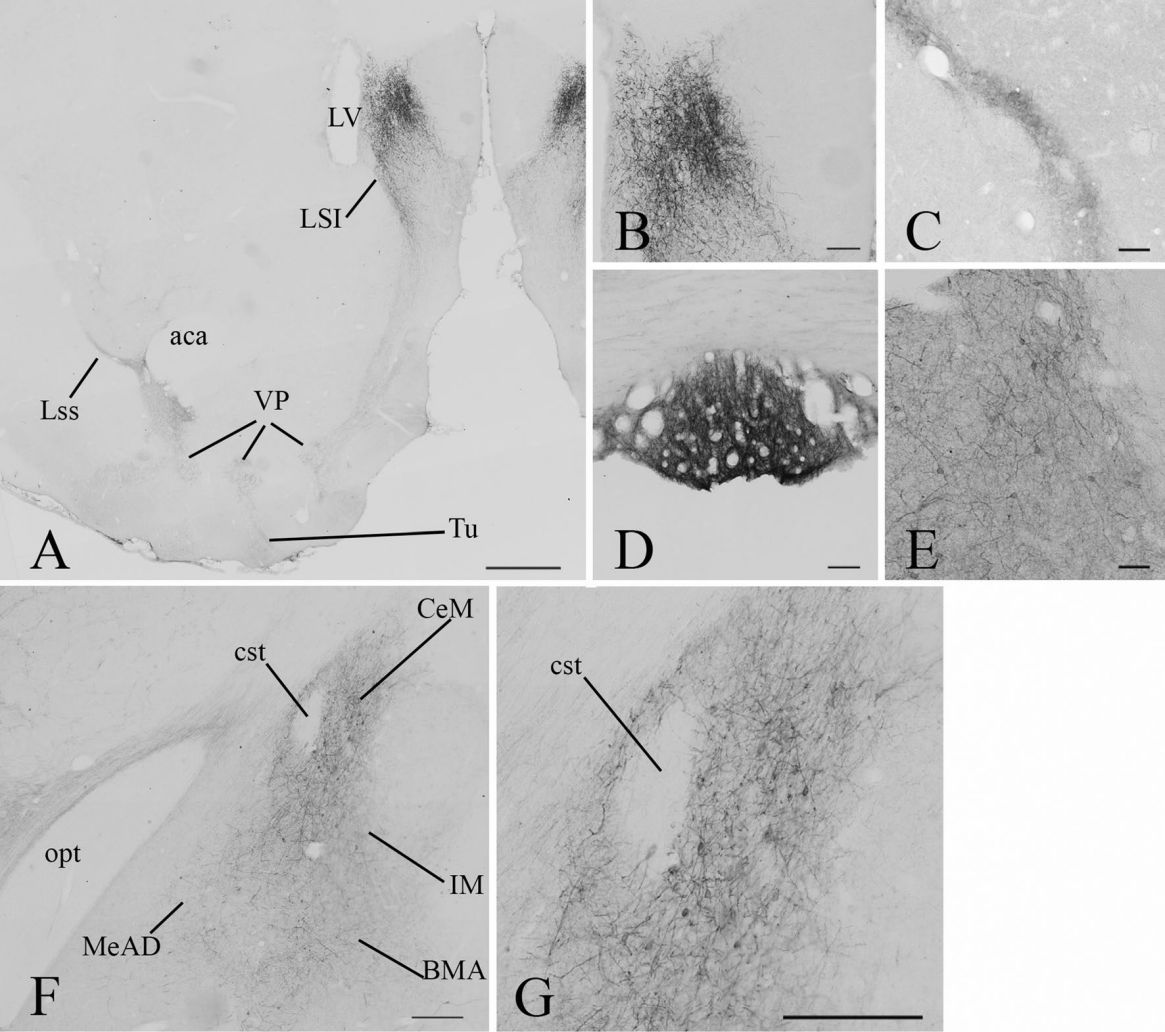
Distribution of GLP-1R-IR elements in the telencephalon. Low magnification image (**a**) illustrates the distribution of GLP-1R-immunoreactivity at the level of the intermediate part of the lateral septal nucleus (LSI) and the ventral pallidum (VP). Very dense network of GLP-1R-IR processes is observed in the LSI. Dense network of GLP-1R-IR fibers is also present in the VP and the lateral stripe of striatum (Lss), but the level of immunoreactivity is slightly lower than in the LSI. Lower intensity GLP1R-IR signal is observed in processes in the olfactory tubercle (Tu). Higher magnification images illustrate the GLP-1R-immunoreactivity in the LSI (**b**) and LSS (**c**). Very high level of GLP-1R-immunoreactivity is present in the subfornical organ (**d**). Large number of GLP-1R-IR perikarya and processes are observed in the bed nucleus of stria terminalis (BNST, **e**). Low magnification image illustrates the GLP-1R-mmunoreactivity in the amygdala (**f**). Large number of GLP-1R-IR perikarya and fibers are present in the medial part of the central amygdaloid nucleus (CeM), while less immunoreaktive perikarya and somewhat less dense fiber network are observed in the anterodorsal part of the medial amygdaloid nucleus (MeAD), the anterior part of the basomedial amygdala (BMA) and the main intercalated amygdaloid nucleus (IM). Higher magnification image (**g**) illustrates the GLP-1R-IR elements in the CeM. *aca* anterior commissure, *cst* commissural stria terminalis, *LV* lateral ventricle, *opt* optic tract

High or moderate density of GLP-1R-IR elements were detected in the dorsal tenia tecta (fibers), olfactory tubercle (fibers and perikarya) (Figs. 3a and 4), lateral septal nuclei (Fibers and some perikarya), lateral stripe of the striatum (fibers) (Figs. 3b–e and 4) and bed nucleus of the stria terminalis (fibers and perikarya) (Figs. 3d, g and 4). In the basal ganglia, low to moderate density of GLP-1R-IR fibers and cell bodies were detected in the nucleus accumbens shell, caudate putamen (Fig. 3a–g), globus pallidum (Fig. 3f, h, i) and ventral pallidum (Fig. 3b–f). High number of GLP-1R-IR cell bodies and fibers were observed in parts of the amygdala including the medial and central amygdaloid nucleus and sublenticular extended amygdala (Figs. 3h–j and Fig. 4). Within the amygdala, somewhat lower density of GLP-1R-IR elements was observed in the anterior part of the basomedial amygdala and in the intercalated amygdaloid nucleus.

Very low level of immunoreactivity was observed in scattered perikarya in cortical areas and in the granular layer of the dentate gyrus. In these two areas, the density and the pattern of immunoreaction were highly variable among the studied brains.

#### Distribution of the GLP-1R-immunoreactivity in the diencephalon

**Fig. 5:**
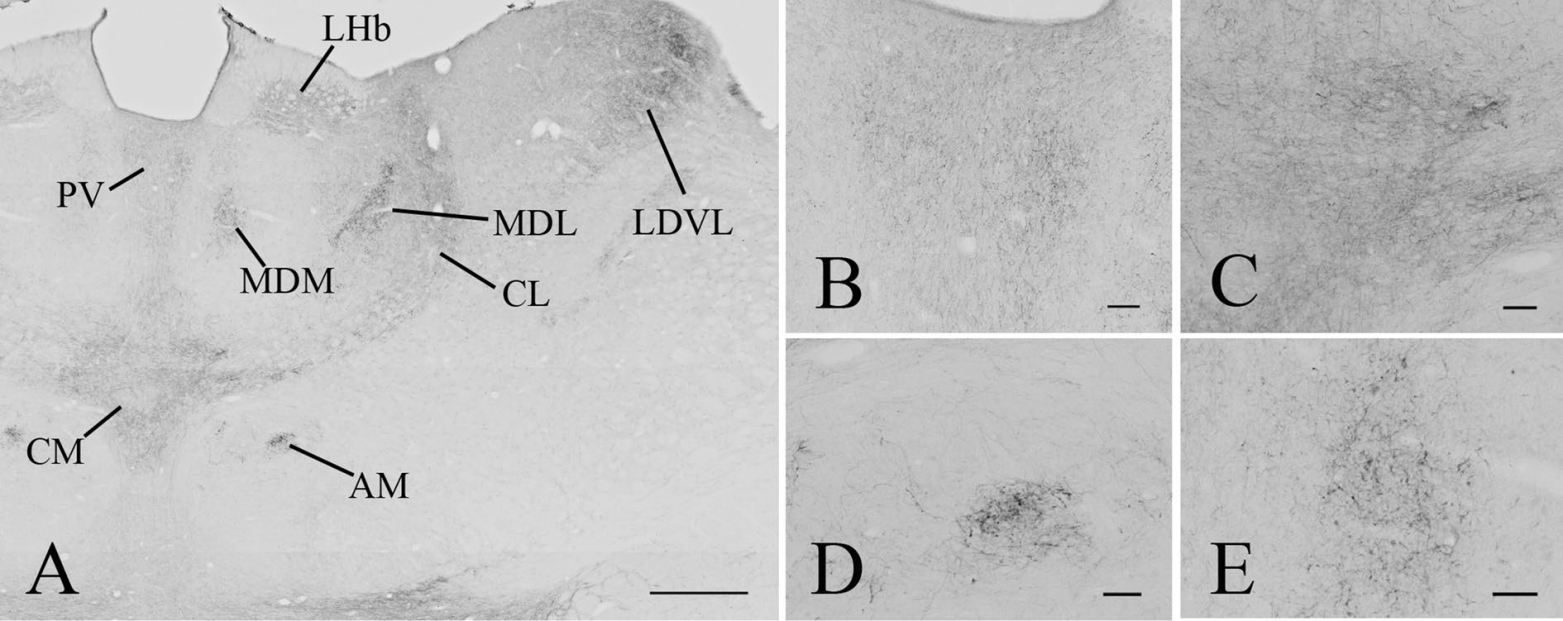
Distribution of GLP-1R-immunoreactivity in the thalamus. Low magnification image illustrates the distribution of GLP-1R-IR elements in the thalamus (**a**). Dense network of GLP-1R-IR fibers can be seen in the paraventricular (PV), central medial (CM), centrolateral (CL), medial (MDM) and lateral parts of the mediodorsal (MDL) thalamic nuclei, the anteromedial thalamic nucleus (AM), the ventrolateral part of the laterodorsal thalamic nucleus (LDVL and in the lateral habenular nucleus. Higher magnification images show the GLP-1R-IR elements in the PV (**b**), CM (**c**), AM (**d**) and MDM (**e**). Scale bars are 500 µm on (**a**) and 50 µm on (**b**–**e**)

A large number of thalamic nuclei also contained a dense network of GLP-1R positive fibers (Fig. 5), but the level of immunoreactivity was lower than in the previously described brain regions nuclei (Fig. 3h–k). GLP-1R-IR cell bodies were observed in the paratenial, parafascicular and ethmoid thalamic nuclei. The GLP-1R-IR fibers had more widespread distribution in the thalamus. Dense networks of GLP-1R fibers were observed in the interanterodorsal, paraventricular, paratenial and reuniens thalamic nuclei and in the ventral lateral geniculate nucleus. Less dense fiber networks were observed in the mediodorsal, anteromedial, anteroventral, lateroposterior, laterodorsal, central medial, centrolateral, parafascicular, posterior limitans and reticular thalamic nuclei (Fig. 3h–k).

In the epithalamus and subthalamus, a dense network of GLP-1R-IR fibers were detected in the zona incerta and the lateral habenular nucleus, while sparser fiber networks were observed in the subincertal, subthalamic and subgeniculate nuclei (Fig. 3i–k). In addition to fibers, the zona incerta also contained GLP-1R-IR cell bodies (Fig. 3i, j).

**Fig. 6:**
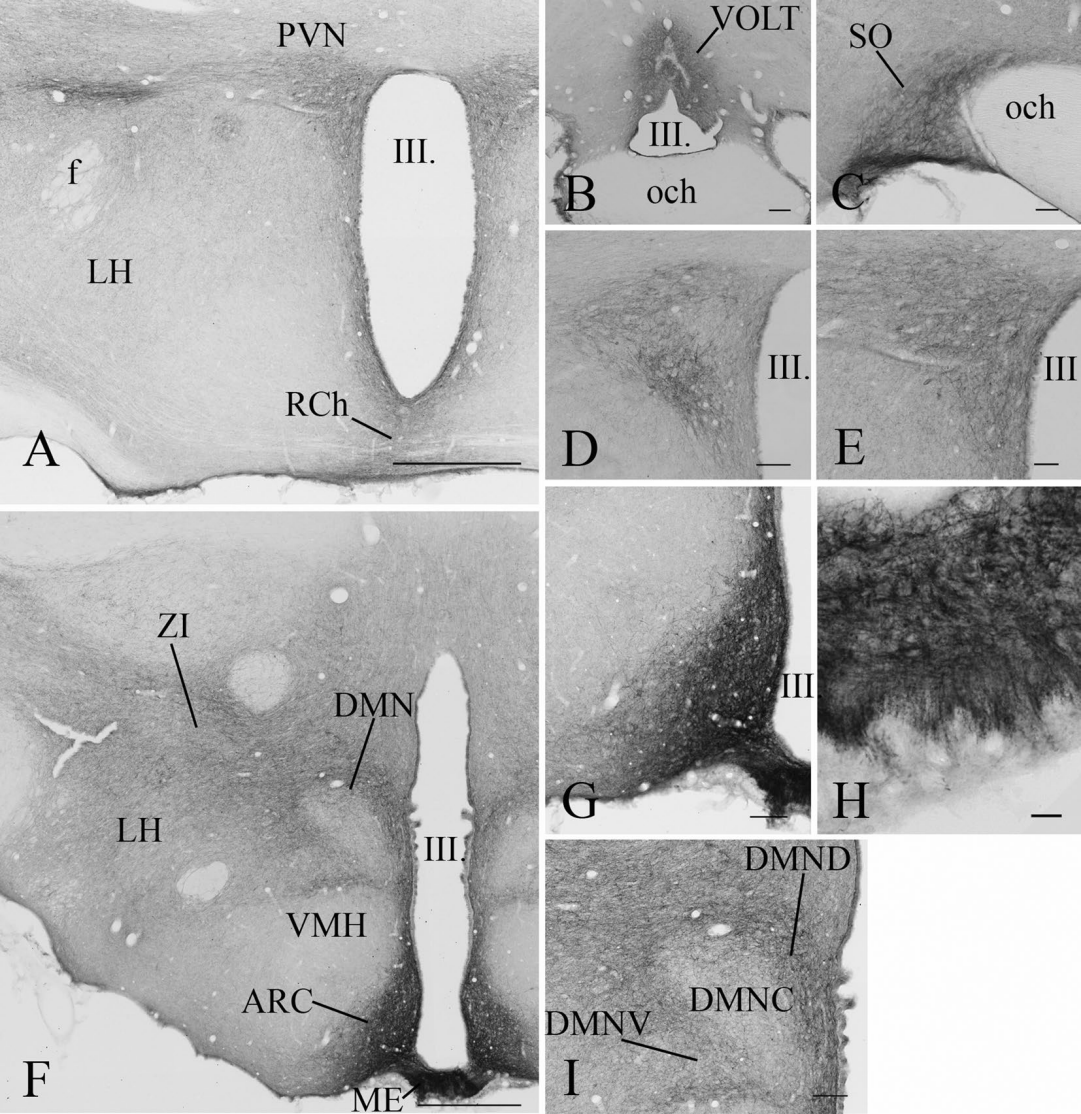
Distribution of the GLP-1R-immunoreactivity in the hypothalamus. A low magnification image (**a**) illustrates the distribution of GLP-1R-immunoreactive profiles at the level of the hypothalamic paraventricular nucleus (PVN). Dense network of GLP-1R-IR fibers can be seen in the PVN, the periventricular area and in the retrochiasmatic area (RCh). Less dense fiber network can be also seen in the lateral hypothalamus (LH). GLP-1R-IR perikarya is present in the PVN. Dense network of GLP-1R-IR fibers is also present in the vascular organ of laminae terminalis (VOLT) (**b**). The supraoptic nucleus (SO) (**c**) is filled with GLP-1R-IR processes, while both GLP-1R-IR perikarya and fibers are present in the mid (**d**) and caudal levels (**e**) of the PVN. Low magnification image (**f**) illustrates the distribution of the GLP-1R-IR profiles at the level of hypothalamic dorsomedial nucleus (DMN) and the arcuate nucleus (ARC). Note the very high density of the GLP-1R-immunoreactivity in the ARC and the median eminence (ME). Dense network of GLP-1R-IR profiles is also present in the DMN, zona incerta (ZI) and in the LH. GLP-1R is absent from the ventromedial hypothalamic nucleus (VMH). Higher magnification images demonstrate the presence of the GLP-1R-IR structures in the ARC (**g**), the ME (**h**) and in the DMN (**i**). Within the DMN, dense fiber network is present in the ventral (DMNV) and dorsal (DMND) parts of the nucleus, while the density of immunoreactive fibers is much lower in the compact part of the nucleus (DMNC). *III* Third ventricle, *f* fornix, *och* optic chiasm. Scale bars are 500 µm on **a** and **f**; 100 µm on **d**, **g**, **i**, 50 µm on **c**, **e** and 20 µm on **h**

GLP-1R-IR cell bodies and fibers also had widespread distribution in the hypothalamus (Fig. 6). The density of the GLP-1R protein-containing profiles was the highest in the ARC, the hypothalamic paraventricular nucleus (PVN) (Fig. 3g–i), supraoptic nucleus, hypothalamic dorsomedial nucleus (DMN) and in the median eminence (ME) (Fig. 3f–i). Dense GLP-1R-immunoreactivity was also observed in the medial and lateral mammillary nuclei and in the supramammillary nucleus (Fig. 3k). Moderate density signal was detected in the preoptic and the lateral hypothalamic areas. In contrast to the surrounding areas and nuclei, lack of GLP-1R protein was observed in the ventromedial hypothalamic nucleus (Fig. 3i, j).

#### Distribution of GLP-1R in the brainstem

**Fig. 7:**
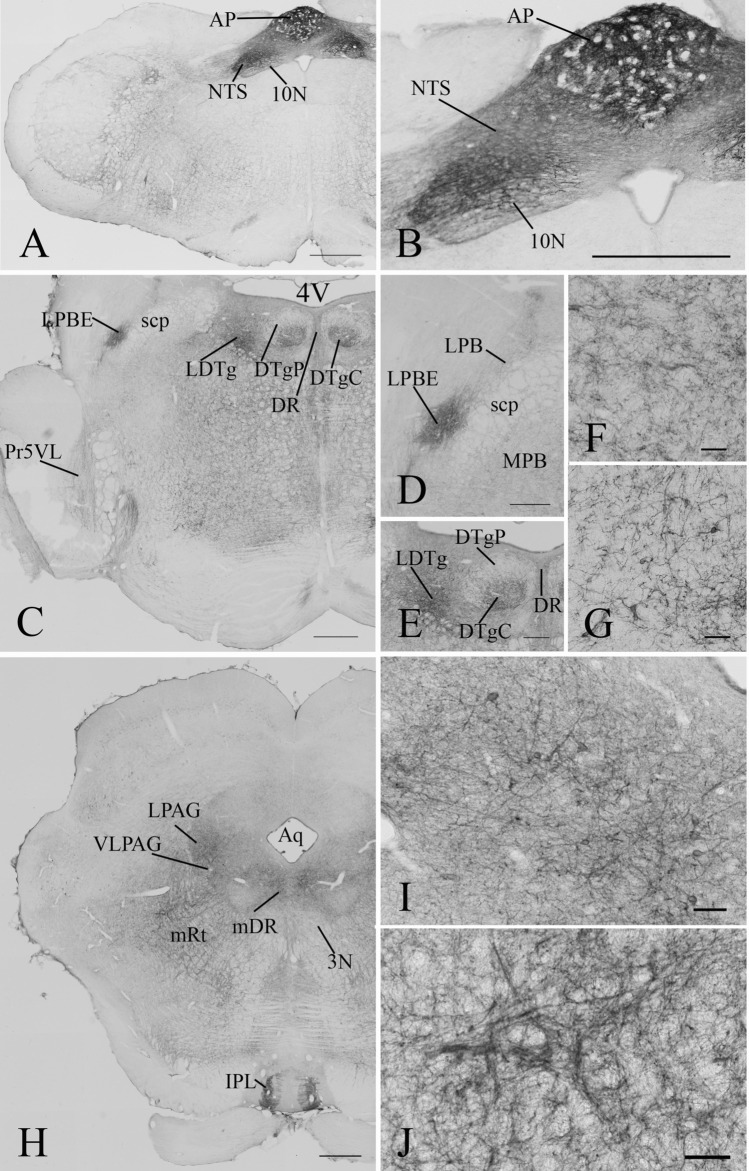
Distribution of GLP-1R in the brainstem. At the level of the medulla (**a**) very high level of GLP-1R-immunoreactivity is present in the area postrema (AP) and the the nucleus of the solitary tract (NTS). GLP-1R-immunoreactivity can also be observed in the dorsal motor nucleus of the vagus (10 N) and in the reticular nuclei. Higher magnification image (**b**) illustrates the GLP-1R-immunoreactivity in the AP, NTS and 10 N. In the pons (**c**), widespread distribution of the GLP-1R-IR profiles was observed at the level of the parabrachial nucleus (PB). This nucleus is also shown at higher magnification (**d**). GLP-1R-IR fibers are observed in both the medial (MPB) and lateral parts (LPB) of the nucleus, however, the external part of the LPB (LPBE) contains a very dense network of GLP-1R-IR fibers. In the dorsal pontin tegmentum, shown at higher magnification at (**e**), high density of GLP-1R-IR fibers is present in the laterodorsal tegmental nucleus (LDTg). High number of GLP-1R-IR perikarya is observed in the central part of the dorsal tegmental nucleus (DTgC), while only GLP-1R-IR fibers are seen in the dorsal part of the nucleus (DTgP). The dorsal raphe (DR) also contains GLP1R-IR fibers. GLP-1R-IR fibers and scattered perikarya can be seen in the reticular formation and the ventrolateral part of the principal sensory trigeminal nucleus (Pr5VL). Higher magnification images of the caudal part of the pontin reticular nucleus (PNC) and the ventral part of the subcoerules (SubCV) are shown on (**f**) and (**g**), respectively. Low magnification image (**h**) illustrates the GLP-1R-IR elements in the mesencephalon at the level of the oculomotor nucleus (3 N). High level of GLP-1R-immunoreactivity is observed in the lateral part of the interpeduncular nucleus (IPL). Dense network of GLP-1R-IR fibers is present in the lateral (LPAG) and ventrolateral (VLPAG) periaqueductal gray, in the mesencephalic dorsal raphe (mDR) and the mesencephalic reticular formation (mRt). GLP-1R-immunoreactive fibers are also present in the oculomotor nucleus, but in much lower density. In addition to fibers GLP-1R-IR perikarya are also present in the LPAG, VLPAG, mDR and mRt. Higher magnification images show the GLP-1R-IR elements in the DR (**i**) and mRt (**j**). *4V* 4th ventricle, *Aq* cerebral aqueductus, *scp* superior cerebellar peduncle Sc. Scale bars are 500 µm on **a**–**c** and **h**; 200 µm on **d** and **e**; 50 µm on **f, g**, **i** and **j**

The most widespread distribution of the GLP-1R-IR profiles was observed in the brainstem (Figs. 3l–p and 7). The reticular formation including the nuclei of cranial nerves contained GLP-1R-IR fibers and perikarya along the entire brainstem (Fig. 3l–p). The periaqueductal gray also contained low to moderate levels of GLP-1R-IR fibers. Some parts of the periaqueductal gray including the lateral periaqueductal area also contained GLP-1R-IR perikarya. Within the mesencephalon, the highest density of GLP-1R-immunoreactivity was observed in the lateral subnucleus of the interpeduncular nucleus (Fig. 3m). GLP-1R-IR fibers were detected in all parts of the dorsal raphe nucleus, but GLP-1R-IR perikarya were observed only in the dorsal part of this nucleus (Fig. 3m). In the substantia nigra, GLP-1R-IR fibers were found only in the medial tier of the compact part, while immunoreactive perikarya were observed in the reticular part of the nucleus (Fig. 3l). In the tectum, low level of GLP-1R-immunoreactivity was only observed in the optic nerve layer and intermediate white layer of the superior colliculus (Fig. 3l, m). In the pons, the highest density of GLP-1R-IR fibers was detected in the external part of the lateral parabrachial nucleus (LPBE) (Fig. 3n). The other parts of the parabrachial nucleus also contained GLP-1R-IR fibers, but in lower density than the LPBE. The dorsal tegmental nucleus the laterodorsal tegmental nucleus, the β part of the central gray, the raphe magnus and the caudal part of the dorsal raphe nucleus also contained high density of GLP-1R-IR fibers (Fig. 3n, o). Especially high density of GLP-1R-IR perikarya were detected in the central part of the dorsal tegmental nucleus, but GLP-1R-IR perikarya were also detected in the caudal part of the dorsal raphe nucleus, dorsomedial tegmental area, ventral part of the subceroleus nucleus, the ventrolateral part of the principal sensory trigeminal nucleus (Fig. 3n).

The highest level of GLP-1R-immunoreactivity was observed in the medulla, in the area postrema where numerous perikarya were observed in a very dense network of fibers (Fig. 3p). In the close proximity of the area postrema, the high density of GLP-1R-IR fibers was observed in the NTS. In addition, a dense fiber network was observed in the raphe pallidus and in the nuclei of the inferior olive.

#### Cerebellum

**Fig. 8:**
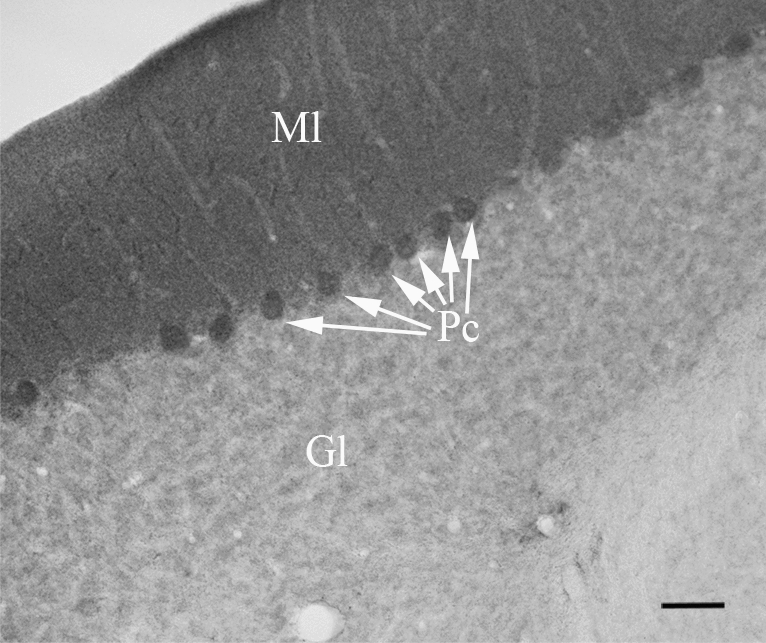
Distribution of GLP-1R-immunoreactivity in the cerebellum. Punctuate GLP-1R-immunoreactivity labels the cell bodies of Purkinje cells (arrows) and the entire molecular layer. Low level of punctuate GLP-1R-immunoreactivity is also present in the granular layer. *Gl* granular layer, *Ml* molecular layer, *Pc* Purkinje cells; scale bar = 50 µm

In the cerebellum, dense GLP-1R-immunoreactivity with punctuate appearance filled the cell bodies of the Purkinje cells and the entire molecular layer (Fig. 8). Very low levels of punctuate GLP-1R-immunoreactivity was observed in the granular layer.

#### Ultrastructural localization of the GLP-1R-immunoreactivity

**Fig. 9:**
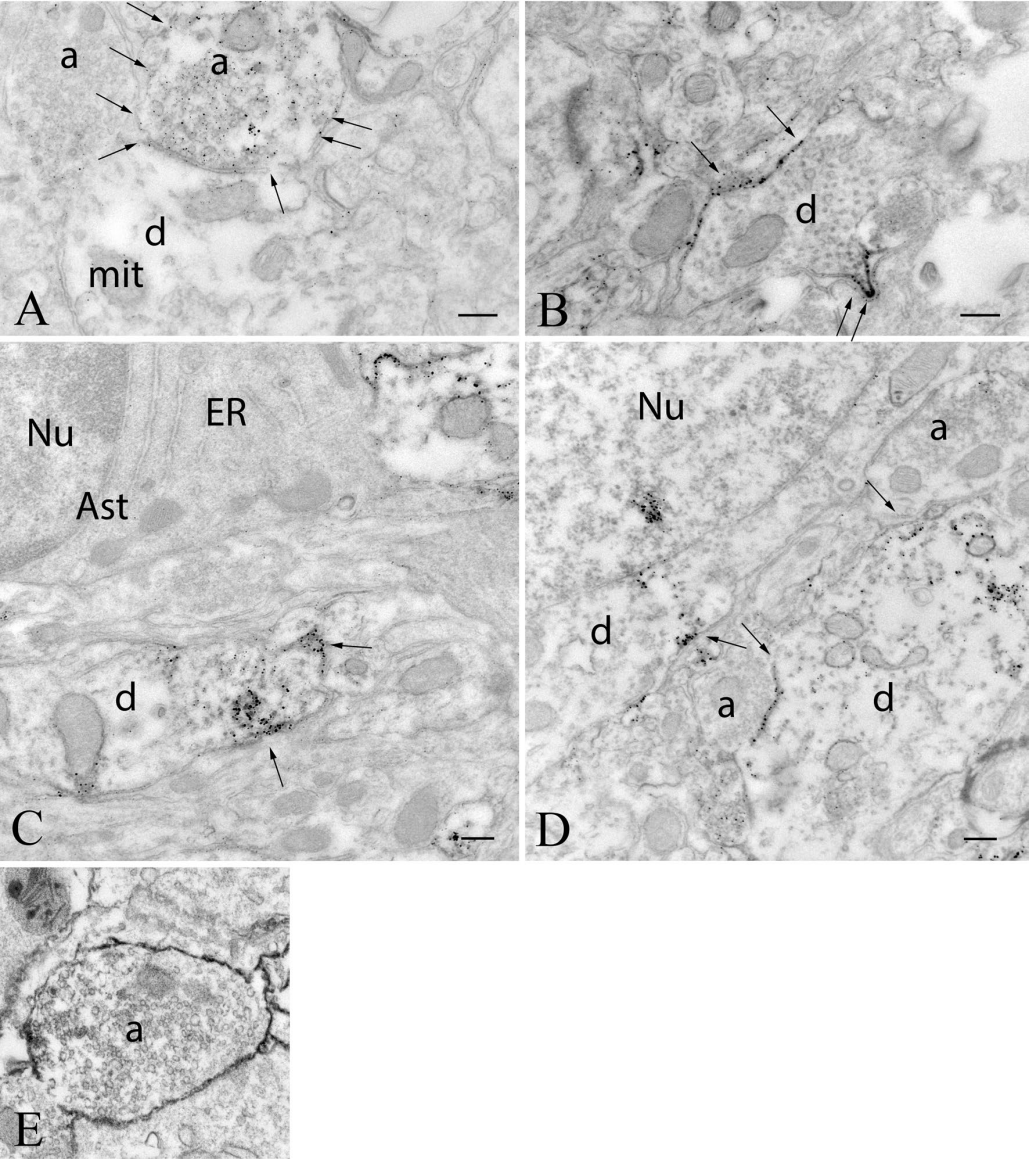
Ultrastructural localization of GLP-1R in the arcuate nucleus. GLP-1R-immunoreactivity is labeled with electron-dense silver particles. In this nucleus of rats, the GLP-1R-immunoreactivity is present in axons (**a**), dendrites (**b**–**d**) and neuronal perikarya (**d**), while GLP-1R-immunoreactivity was not observed in the perikarya of astrocytes (**c**). **a** Illustrates a GLP-1R-immunoreactive axon (arrows) forming symmetric type synapse on an unlabeled dendrite. **b** Demonstrates the presence of silver grains labeling GLP-1R-immunoreactivity on the membrane of a dendrite (arrows). **c** Illustrates a dendrite where GLP-1R is present in both the cytoplasm and on the cell membrane (arrows). The astrocyte shown by this image is unlabeled. **d** Shows GLP-1R-IR perikaryon and dendrite. In both cases, the immuoreactivity is associated to both the cytoplasm and the cell membrane (arrows). **e** Demonstrates that axonal localization of the GLP-1R is also present in the ARC of mice. *a* axon, *Ast* astrocyte, *d* dendrite, *ER* endoplasmatic reticulum, *mit* mitochondrium, *Nu* nucleus, scale bars = 0.25 µm

**Fig. 10:**
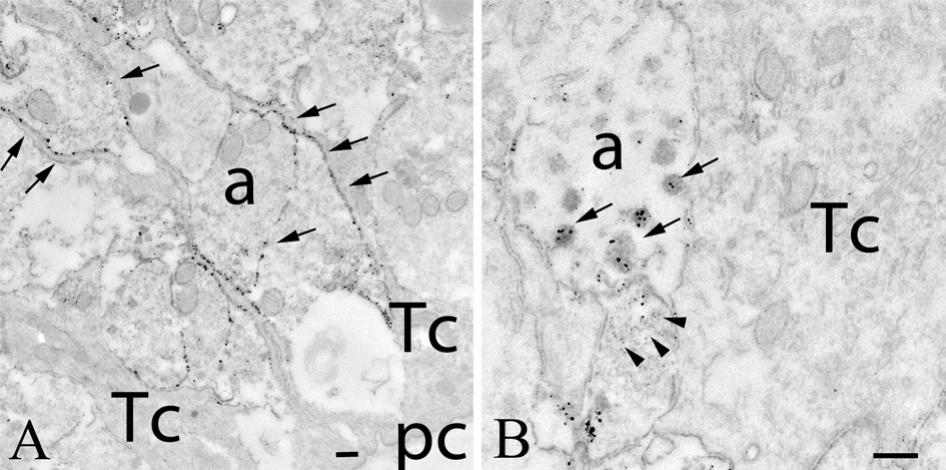
Distribution of GLP-1R-immunoreactivity in the median eminence. In the external zone of the median eminence (**a**), numerous GLP-1R-IR axons (arrows) can be observed. The silver grains denoting the GLP-1R-immunoreactivity encircle the hypophysiotropic axon terminals in the vicinity of the portal capillaries. Silver grains were not observed in association with tanycyte processes. The association of the silver grains to dense core (arrows) and small clear (arrowheads) vesicles can also be observed (**b**). *a* axon, *pc* portal capillary, *Tc* tanycyte. Scale bars = 0.25 µm

**Fig. 11:**
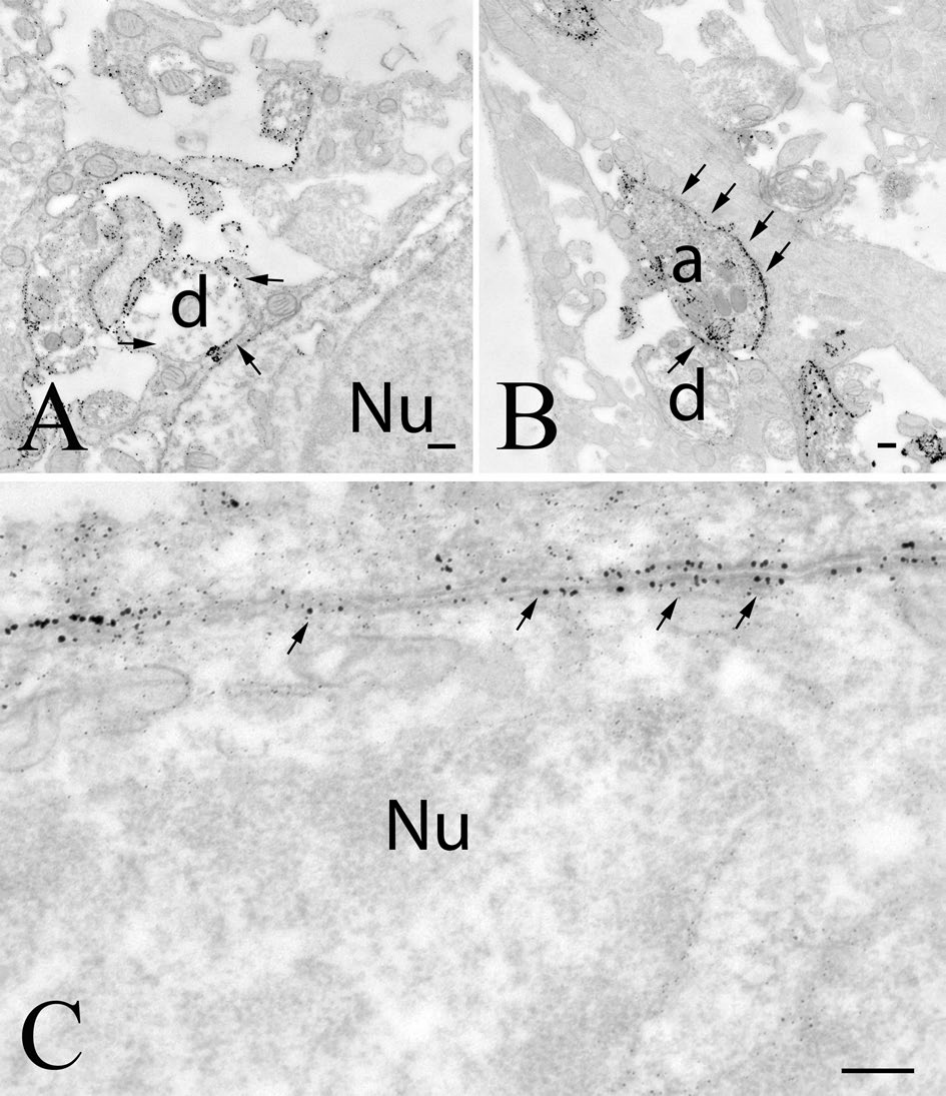
GLP-1R in the area postrema. In this brain area, GLP-1R-immunoreactivity densely labels the surface of dendrites (**a**), axons (**b**) and perikarya (**c**). Arrows point to the silver grains labeling the GLP-1R-immunreactivity. *a* axon, *d* dendrite, *Nu* nucleus; scale bars = 0.25 µm

**Fig. 12:**
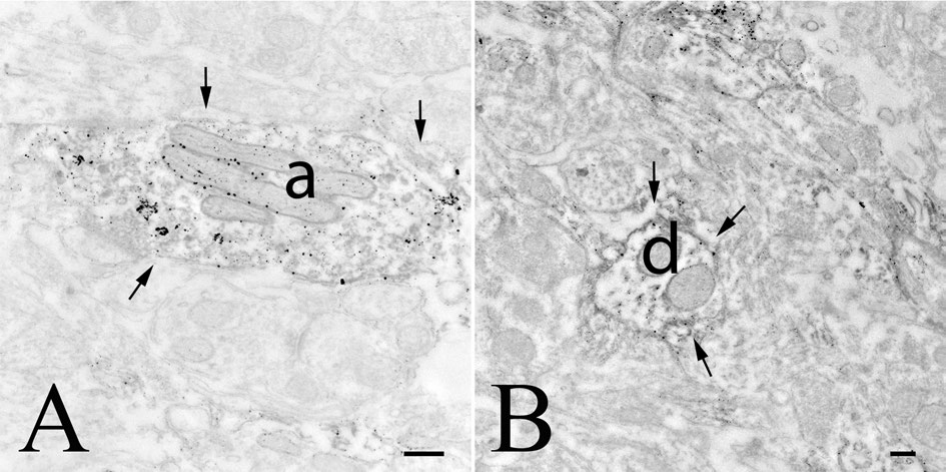
GLP-1R-immunoreactivity in the NTS. In this nucleus, GLP-1R-immunoreactivity was primarily observed in axons (**a**, arrows) and dendrites (**b**, arrows). Note that the silver grains, denoting the GLP-1R-immunoreactivity were predominantly present in the cytoplasm of the labeled structures. *a* axon, *d* dendrite; scale bars = 0.25 µm

To understand the subcellular localization of GLP-1R protein, the ultrastructural examination of GLP-1R-immunoreactivity was performed in the circumventricular organs and the surrounding areas, where the density of the GLP-1R-immunoreactivity was highest, including the ARC (Fig. 9), ME (Fig. 10), AP (Fig. 11) and NTS (Fig. 12). The silver particles labeling the GLP-1R-immunoreactivity were often detected along the membrane of perikarya, dendrites and axons. In the ARC, GLP-1R-immunoreactivity was present in the membrane of the axons (Fig. 9a), dendrites (Fig. 9b–d) and cell bodies (Fig. 9d). In axons, GLP-1R-immunoreactivity was also associated with small, clear, as well as dense core vesicles suggesting that GLP-1R is transported in these vesicles. GLP-1R-immunoreactivity was also associated with axons in the mouse ARC demonstrating that the axonal localization of the receptor is not a species-specific feature (Fig. 9e).

In the external zone of the ME, where the hypophysiotropic axons terminate, GLP-1R-immunoreactivity was associated with the outer surface of numerous axon terminals (Fig. 10a, b). GLP-1R-immunoreactivity was, however, also present in small clear vesicles (Fig. 10a) and in dense-core vesicles (Fig. 10b) of these axons. The density of the GLP-1R-IR hypophysiotropic axon terminals was very high close to the portal capillaries in the external zone (Fig. 10a). GLP-1R-immunoreactivity was not detected in the tanycytes of the ME.

In the AP, silver-gold granules labeling GLP-1R were primarily present on the outer surface of the membrane of axons, dendrites and cell bodies (Fig. 11a–c). In most cases, the GLP-1R-immunoreactivity completely surrounded these profiles.

In the NTS, GLP-1R-immunoreactivity was present in axons (Fig. 12a) and dendrites (Fig. 12b). In the axons, the GLP-1R-immunoreactivity was primarily localized to small clear vesicles. In some cases, the labeled vesicles associated with microtubules of dendrites. In addition, we observed GLP-1R-immunoreactivity in the membranes of labeled processes, but compared to the previously described brain regions, the density of GLP-1R-immunoreactivity was lower in the membranes of axons and dendrites (Fig. 12a, b).

## Discussion

In the current manuscript, we provide a detailed map of the GLP-1R-immunoreactive profiles in the rat brain and describe the subcellular localization of this receptor. In addition to the expected perikaryonal and dendritic localization of the GLP-1R, numerous varicose, axon-like GLP-1R-IR profiles were observed in many parts of the brain. Using immuno-electron microscopy, we demonstrated that indeed, GLP-1R-immunoreactivity was associated not only to perikarya and dendrites but also to axons. GLP-1R was observed in axon terminals forming both symmetric and asymmetric type synapses.

Using peripheral administration of superresolution microscopy compatible GLP-1R antagonist, Ast et al. (2020) also observed ligand binding around the cells of the ARC and AP. However, at the resolutions used, they were unable to distinguish axonal staining. Using electron microscopy to visualize cell membranes, we now provide further information showing that in addition to perikarya and dendrites, the GLP-1R is also present in association to the membranes of axons in these brain regions.

The presence of GLP-1R on axon terminals suggests that GLP-1 signaling may regulate the activity of presynaptic terminals and may modulate both GABA and glutamate release. Since the elevation of intracellular cAMP level is one of the main mediators of GLP-1R signaling (Rowlands et al. 2018; Kawatani et al. 2018; Oride et al. 2017), and cAMP is known to increase the release probability in presynaptic terminals (Chen and Regehr 1997), it is likely that GLP-1 and/or GLP-1 agonists increase the activity of the GLP-1R-containing presynaptic terminals. This hypothesis is supported by the findings of Rebosio et al. (2018) demonstrating that the GLP-1 agonist exendin-4 increases the GABA and aspartate release from hippocampal synaptosomal preparations, and also by our observation demonstrating facilitation of the synaptic input of POMC neurons by GLP-1 signaling (Péterfi 2020). Axonal localization of the receptor was also observed in the mouse brain indicating that the potential presynaptic action of GLP-1 signaling is not a species-specific feature.

At the ultrastructural level, most of the GLP-1R-imunoreactivity was observed in association to extrasynaptic membranes. This localization suggests that the ligand of this receptor is either released extrasynaptically from axons of GLP-1-synthesizing neurons and reach its receptor by volume transmission or it originates primarily from the peripheral blood. As GLP-1R agonists readily enter the ME-ARC and the NTS-AP regions (Ast et al. 2020; Gabery et al. 2020; Secher et al. 2014), it is likely that GLP-1 can also access these brain regions from the circulation and can bind to the extrasynaptic receptors.

We also observed GLP-1R in association with small clear and dense-core synaptic vesicles. The role of the receptor in these vesicles is currently unknown. However, as the GLP-1R is a surface receptor, we hypothesize that GLP-1R is not functioning in these vesicles, rather the receptor is transported in the vesicles to the cell membrane where it can be activated by its extracellular ligand.

We have observed a widespread distribution of the GLP-1R-immunoreactive profiles both in the forebrain and the brainstem. The highest density of the GLP-1R-immunoreactivity was found in circumventricular organs including the area postrema, ME, subfornical organ and the OVLT. The presence of GLP-1R in these brain regions is intriguing. The GLP-1 neurons do not project to these areas (Gu et al. 2013), but the circumventricular organs are located outside of the blood–brain barrier suggesting that GLP-1 of peripheral origin could be the ligand of these receptors. Indeed, binding of peripherally administered GLP-1 to neurons of the subfornical organ and the area postrema has been demonstrated (Orskov et al. 1996). In the external zone of the median eminence, where the axons of hypophysiotropic neurons terminate, GLP-1R-immunoreactivity heavily labels the surface of axon terminals. Thus, it is likely that peripheral GLP-1 or GLP-1R agonists can regulate the release of hypophysiotropic hormones into the portal capillaries and, therefore, may regulate the activity of neuroendocrine axes. In the ME, GLP-1R-immunoreactivity was not observed in the β2 tanycytes. Thus, it is unlikely that these tanycytes are involved in the transport of GLP-1 from the ME to the CSF or to the ARC in the rat. The presence of the receptor, however, was not studied in all subtypes of tanycytes, so we were not able to confirm the expression of GLP-1R in α-tanycytes previously suggested to be involved in the transport of GLP-1R agonists into the ARC in mice (Gabery et al. 2020).

The cells of the blood–brain-barrier free AP can also be accessible for peripheral GLP-1 (Orskov et al. 1996). In the AP, the majority of perikarya and dendrites and numerous axons are almost completely ensheethed by silver grains labeling the GLP-1R-immunoreactivity suggesting that GLP-1 may influence directly the neurons of the area postrema, but it can also influence the input of these neurons. Indeed, it was shown by Kawatani et al. (2018) by patch-clamp electrophysiology that GLP-1 directly depolarizes the neurons of the area postrema and increases their firing. Furthermore, lesioning of area postrema markedly decreases the effect of peripherally administered GLP-1R agonist exendin-4 on the neuronal activation in a number of brain regions like in the ventrolateral medulla, ARC and parabrachial nucleus, indicating that the area postrema mediates the GLP-1 signaling toward important energy homeostasis related brain regions (Baraboi et al. 2010). Intriguingly, however, lesion of the AP does not prevent the inhibitory effect of exendin-4 on the food intake (Baraboi et al. 2010) and also does not prevent the body weight reduction induced by the long acting GLP-1R agonist liraglutide (Secher et al. 2014). These data indicate that the AP mediates central effects of GLP-1, but the role of the AP is not indispensable for the GLP-1 signaling induced regulation of food intake and body weight.

In the vicinity of the area postrema, the NTS is also densely labeled with GLP-1R-immunoreactivity. In this nucleus, the receptor was found in dendrites and in axons suggesting that GLP-1 may have a direct effect on the NTS neurons, but can also modulate the input of these cells. Interestingly, in contrast to other studied brain regions, the GLP-1R-immunoreactivity was primarily observed inside the neuronal profiles and not on their surface. As intra-NTS administration of GLP-1R agonist regulates food intake and hedonic values of food (Richard et al. 2015), the GLP-1R is obviously functional in this nucleus despite its primarily intracellular localization. GLP-1R is known to internalize after ligand binding (Fletcher et al. 2018), thus the high level of intracellular GLP-1R in this nucleus may suggest that the ligand occupancy of the GLP-1R can be relatively high in this nucleus. Although NTS is inside the blood–brain-barrier, peripheral hormones, like leptin, and ghrelin have been shown to influence energy homeostasis by acting directly on NTS neurons (Grill and Hayes 2012) indicating that peripheral hormones, including GLP-1, can enter this nucleus. Alternatively, GLP-1 released from the local proglucagon neurons may also act on the GLP-1R in this nucleus.

Very high levels of GLP-1R-immunoreactivity was present in the ARC, another brain region known to function as a sensor of peripheral energy homeostasis (Morton et al. 2006). Like in the AP, perikaryonal, dendritic and axonal localization of the GLP-1 receptor was observed in this nucleus. Since there are only very few GLP-1 containing axons in the ARC (Larsen et al. 1997), it is likely that the GLP-1R-containing neuronal profiles are primarily regulated by peripheral GLP-1 in this nucleus. Within the ARC, the anorexigenic POMC neurons are known to be regulated by GLP-1 signaling (Secher et al. 2014; Gabery et al. 2020). POMC neurons accumulate the GLP-1R agonist liraglutide after its peripheral administration, furthermore, GLP-1 depolarizes these neurons and increases their firing rate (Secher et al. 2014) suggesting that the effect of GLP-1 and its agonists on the POMC neurons may contribute to the anorexigenic effect of these compounds (Secher et al. 2014). In the ARC, however, far more neurons seem to contain the GLP-1R and/or are innervated by GLP-1R-containing axons, therefore, further studies are needed to understand the chemotype and function of GLP-1 target neurons in this nucleus.

Although circumventricular organs and the neighboring ARC and NTS contain the highest density of GLP-1R, there is a far more widespread distribution of this receptor. The presence of GLP-1R in energy homeostasis related nuclei, like the DMN, PVN and BNST, and in reward regions such as nucleus accumbens and ventral pallidum is in accordance with the known function of GLP-1 in the regulation of reward processes (Hayes and Schmidt 2016).

GLP-1 receptors are also observed in regions that are not directly related to the regulation of energy homeostasis such as thalamic nuclei, lateral septum and reticular nuclei of the brainstem suggesting that GLP-1 signaling has much more diverse functions in the brain.

Despite the fact that GLP-1 effect has been detected in the hippocampus and cortex (Hsu et al. 2015; Csajbok et al. 2019), low level of GLP-1R-immunoreactivity with variable pattern and density was observed in these areas, therefore, further studies with more sensitive antibodies and/or methods are necessary to elucidate the distribution of GLP-1R protein in hippocampal and cortical areas. The very low level of GLP-1R-immunoreactivity in the rat hippocampus is in contrast to the relatively high level of GLP-1R-immunoreactivity in the mouse hippocampus (Jensen et al. 2018) suggesting a strong species difference of the GLP-1R signaling in this brain region.

In contrast to these brain regions, GLP-1R-immunoreactivity very strongly labeled the Purkinje cells and the molecular layer of the cerebellum suggesting that the GLP-1 signaling may have an important effect in the regulation of cerebellar function.

The described distribution of GLP-1R-immunoreactivity is highly similar to the distribution of GLP-1 and exendin-4 binding sites observed by Goke et al. (1995) using unfixed sections of rat brain. They found the highest receptor binding in the lateral septum, in the circumventricular areas and in regions located around the circumventricular areas where we found the highest density of receptor immunoreactivity. In addition, they also did not observe binding in cortical areas and the hippocampus, where we observed only very faint immunoreaction signal that could not be unequivocally differentiated from the background signal.

The observed distribution of the GLP-1R-IR perikarya is also similar to the distribution of the GLP-1R mRNA expressing neurons (Merchenthaler et al. 1999). A major difference is, however, that widespread distribution of GLP-1R mRNA was observed in the hippocampus of rats (Merchenthaler et al. 1999), while we observed only very faint immunoreaction signal in this brain area. We do not know whether the GLP-1R protein content of these cells is very low despite their readily detectable GLP-1R mRNA content, or if the protein is transported to axons that terminate outside the hippocampus. Alternatively, a special variant of the receptor could be expressed in the hippocampus that is not detected by the used antibody. The latter explanation, however, is unlikely due to the very minimal GLP-1 binding in the hippocampus.

The distribution of GLP-1R-IR profiles in the mouse brain was earlier described by Jensen et al. (2018). That study, however, focused primarily on the distribution of GLP-1R-IR perikarya and described the presence of GLP-1R-IR fibers only in a few brains areas. In contrast, we observed a very widespread distribution of GLP-1R-IR fibers in the brain and using immuno-electron microscopy, we proved that these fibers are not only dendrites but GLP-1R also has presynaptic localization. As we also observed a very dense network of GLP-1R-IR fibers in the mouse brain and proved the presence of the receptor in axons of mouse hypothalamus, we believe that this discrepancy of the two description is not simply due to species differences. Jensen et al. (2018) performed their immunostaining on paraffin-embedded tissues of mice perfused with 4% PFA and used diaminobenzidine as chromogen. We did not test the effect of paraffin embedding, but in our preliminary studies, we observed that the addition of 1% acrolein to the PFA solution highly improves the detection of GLP-1R with the used antibody. Therefore, we used this fixation method in our study. In addition, we used Ni-DAB developer that can also increase the sensitivity of immunocytochemistry compared to the use of diaminobenzidine.

In summary, we demonstrated a very widespread distribution of GLP-1R protein in the rat brain with the highest levels in the circumventricular organs and in the ARC and NTS. We demonstrated that in addition to the expected perikaryonal localization, GLP-1R is also present in axons suggesting that GLP-1 not only has direct effects on target neurons, but it can also regulate the presynaptic input of neuronal populations and may regulate neuroendocrine axes by influencing the activity of hypophysiotropic terminals.

## Supplementary Information

Below is the link to the electronic supplementary material.

Supplementary file1 (PDF 352 KB)

## Data Availability

(Data transparency) All data associated with this study are present in the paper.

## Code availability

(Software application or custom code) not applicable.
