# Supplementary material for: Distribution and ultrastructural localization of the glucagon-like peptide-1 receptor (GLP-1R) in the rat brain
Source: Brain Struct Funct. 2020 Dec 20;226(1):225–45. doi: 10.1007/s00429-020-02189-1 (PMC7817608; doi:10.1007/s00429-020-02189-1)
Supplement: Supplementary file 1 — Supplementary file1 (PDF 352 KB) [file 429_2020_2189_MOESM1_ESM.pdf]

Supplementary Figure

Distribution and ultrastructural localization of the glucagon like peptide-1 receptor (GLP-1R) in the rat brain

Erzsébet Farkas, Anett Szilvásy-Szabó, Yvette Ruska, Richárd Sinkó, Morten Grønbech Rasch, Thomas Egebjerg, Charles Pyke, Balázs Gereben, Lotte Bjerre Knudsen, Csaba Fekete

Journal Name: **Brain Structure and Function**

Corresponding Author:

Csaba Fekete Md, PhD

Laboratory of Integrative Neuroendocrinology

Institute of Experimental Medicine,

1083 Budapest, Hungary

Email: fekete.csaba@koki.hu

Rabbitization of the monoclonal anti-GLP-1R antibody

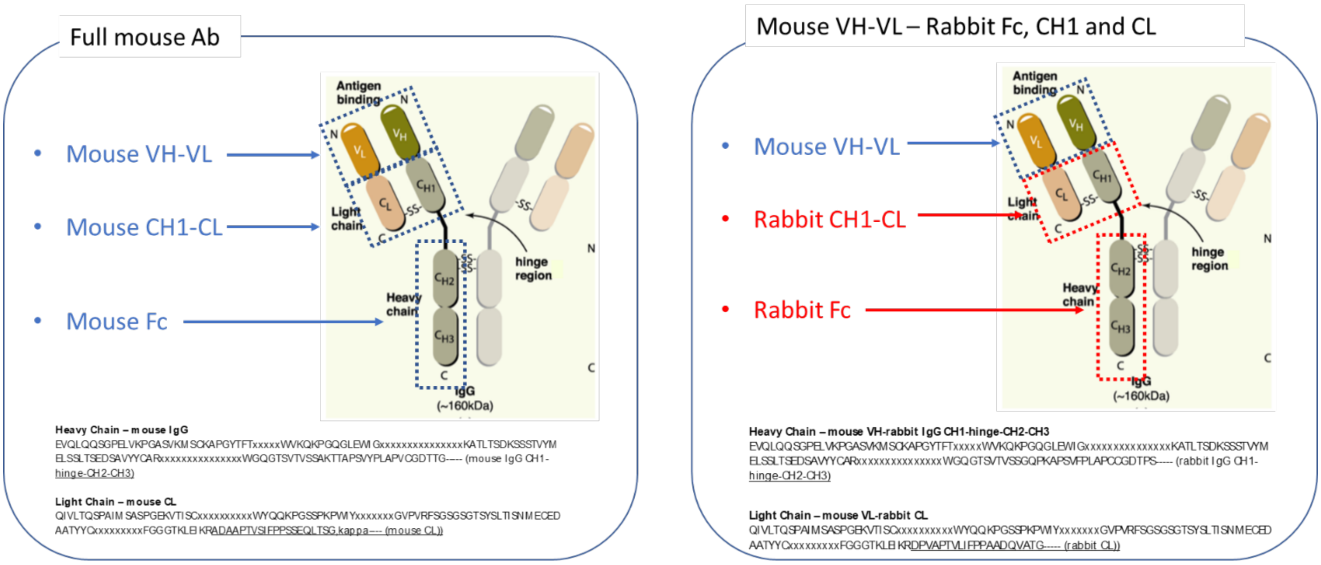

Supplementary Figure 1. Grafting of mouse VH-VL (variable domains of heavy and light chain) sequences raised against GLP-1R onto rabbit CH1-CL

In order to avoid the background signal caused by the presence of endogenous mouse IgG in tissues, the variable domains of mouse monoclonal anti-GLP-1R antibody clone 7F38A2 were grafted onto rabbit IgG constant domains. The procedure were performed in a similar way as desribed by Bhatti et al. (Bhatti et al. 2019). In short, the variable mouse sequences from mouse monoclonal anti-GLP-1R antibody clone 7F38A2 were cloned into rabbit IgG1 heavy chain and rabbit lambda light chain vectors and expressed transiently in HEK 293 cells before Protein A purification.

Reference

Bhatti MM, Cai AG, Theunissen JW (2019) Binding affinities of human IgG1 and chimerized pig and rabbit derivatives to human, pig and rabbit Fc gamma receptor IIIA. PloS one 14 (7):e0219999. doi:10.1371/journal.pone.0219999
